# Effects of textured insoles and elastic braces on dynamic stability in patients with functional ankle instability

**DOI:** 10.1186/s13047-023-00662-8

**Published:** 2023-09-13

**Authors:** Yunqi Tang, Xinyue Li, Yi Li, Peiyao Liang, Xinyu Guo, Cui Zhang, Pui Wah Kong

**Affiliations:** 1 College of Art and Design, Shaanxi University of Science and Technology, Xi ’an, 710021 China; 2 Department of Foot and Ankle Surgery, Honghui Hospital Affiliated to Medical College of Xi ’an Jiaotong University, Xi ’an, 710054 China; 3 Sport biomechanics lab, Shandong Institute of Sports Science, Jinan, 250014 China; 4 Graduate School of Shandong Physical Education University, Jinan, 250014 China; 5 Physical Education and Sports Science Academic Group, National Institute of Education, Nanyang Technological University, Singapore, 637616 Singapore

**Keywords:** Injury, Rehabilitation, Textured insole, Ankle brace, Functional ankle instability, Dynamic stability

## Abstract

**Background:**

Functional ankle instability (FAI) is a common condition that affects individuals who have experienced previous ankle sprains. Textured insoles and elastic ankle braces have been previously used as interventions to improve stability in FAI patients. However, the optimal combination of these interventions has not been fully explored. The objective of this study was to investigate the effects of different types of textured insoles and elastic ankle braces on the dynamic stability of individuals diagnosed with FAI.

**Methods:**

The study involved 18 FAI patients who performed single-leg landing tasks with and without wearing an eight-band elastic ankle brace while wearing textured insoles with protrusion heights of 0 mm, 1 mm, and 2 mm. The dynamic posture stability index (DPSI) and its components in the anterior-posterior (APSI), mediolateral (MLSI) and vertical (VSI) directions were calculated from the ground reaction force collected from the Kistler force plate during the first three seconds of the landing tasks.

**Results:**

A significant interaction was found between textured insole type and ankle brace for DPSI (*P* = 0.026), APSI (*P* = 0.001), and VSI (*P* = 0.021). However, no significant interaction was observed for MLSI (*P* = 0.555). With elastic ankle braces, textured insoles with 1-mm protrusions significantly enhanced anterior-posterior, mediolateral, vertical, and overall stability compared to textured insoles with no and 2 mm protrusions (*P* < 0.05). Without elastic ankle braces, textured insoles with 1-mm protrusions significantly improved the anterior-posterior (*P* = 0.012) and overall stability (*P* = 0.014) of FAI patients compared to smooth insoles.

**Conclusions:**

The combination of textured insoles with 1-mm protrusion heights and an elastic ankle brace could enhance the dynamic stability of individuals with FAI, potentially mitigating the risk of ankle sprains.

## Background

Ankle sprains are among the most common foot-ankle injuries and are frequently encountered in sports [1]. Unfortunately, up to 70% of patients who experience a first ankle sprain will continue to experience symptoms and recurrent injuries [2]. Furthermore, approximately 15-20% of individuals with acute ankle sprains will develop chronic ankle instability (CAI) [3]. Functional ankle instability (FAI) is a subtype of CAI characterized by symptoms such as recurrent ankle sprains, pain, swelling [4–6], and impaired dynamic postural control [7]. FAI can significantly impact an individual’s ability to participate in daily activities and sports, highlighting the importance of appropriate treatment for improving quality of life and preventing further injuries.

Foot orthoses, including orthopedic insoles, are a common therapeutic intervention for individuals with FAI due to their versatility, convenience, and cost-effectiveness [8]. Previous studies have demonstrated that foot orthoses can improve the postural stability of FAI patients by providing proprioception and neuromuscular stimulation [2, 9]. A study by Haddadi et al. [2] revealed that semi-rigid and soft orthoses improved the dynamic stability of FAI patients after a 4-week treatment period. Similarly, Abbasi et al. [9] found that orthopedic insoles with textured surfaces can increase the reach distance in the star excursion balance test for FAI patients, implying that foot orthoses are associated with balance improvement in patients with FAI. The protrusion heights of textured insoles may affect the degree of plantar stimulation and proprioceptive feedback, which are important factors for dynamic stability. It has been proposed that an optimal sensory threshold exists for plantar stimulation for enhanced postural balance, but it remains unclear what the threshold value is [10]. Therefore, it is of interest to investigate the effect of different protrusion heights of textured insoles on the dynamic stability of individuals with FAI.

Besides orthopedic insoles, ankle braces are commonly used in the treatment of FAI patients to improve stability and prevent reinjury [11, 12]. An elastic ankle brace is a type of ankle support that provides compression and flexibility to the ankle joint without restricting its normal range of motion. John et al. [11] found that using elastic ankle braces was ineffective in improving dynamic balance, while Haddadi et al. [12] found that the combination of ankle support and insoles improved postural control and increased proprioception. To the best of our knowledge, the effect of combining elastic ankle braces and textured insoles with different protrusion heights on FAI patients’ dynamic stability is unclear.

The drop landing task is a common method to assess dynamic stability in individuals with FAI, as it simulates a high-risk situation for ankle sprains [13, 14]. The drop landing task involves dropping from a platform onto a force plate without initial vertical velocity and maintaining a single-leg stance for a certain period of time [15]. The ground reaction force data collected from the force plate can be used to calculate various stability indices that reflect the ability of the individual to control their body motion during landing [15]. Drop landing tasks also have the advantages of being simple, safe, and easy to perform compared to other methods, such as hopping or cutting tasks [14]. Therefore, the purpose of this study was to investigate the effects of textured insoles with different protrusion heights and elastic ankle braces on dynamic postural stability in individuals with FAI during drop landing tasks.

The study has four hypotheses: (a) Wearing elastic ankle braces would significantly improve the dynamic stability of FAI patients compared with no ankle braces; (b) Dynamic stability would be better when participants wear textured insoles with higher protrusions than those with lower protrusions; (c) The combination of textured insoles and elastic ankle braces would lead to a greater improvement in dynamic stability when compared to using either textured insoles or elastic ankle braces alone. (d) Ankle braces and textured insoles could enhance the perceived stability and comfort in individuals with FAI. The outcomes of this research will offer valuable insights into enhancing dynamic postural stability and thus support the development of effective rehabilitation strategies for individuals with FAI.

## Methods

This study used a cross-sectional study design. The sample size of this study was calculated using G * Power (version 3.0.10) [16]. It was based on a two-way repeated-measures ANOVA with an *f* = 0.30 effect size derived from pre-experimental results. A minimum of 14 participants were needed to achieve 80% statistical power (*α* = 0.05). To account for potential drop-out and technical errors during the experiment, the study involved 18 FAI patients, which consisted of 8 males and 10 females.

This study only included participants who met the following criteria: (a) they reported experiencing ankle instability, loss of control, or leg weakness during certain activities; (b) their score on the Cumberland Ankle Instability Tool (CAIT) was 24 or lower [17, 18]. The CAIT can discriminate between subjects with and without functional ankle instability. The CAIT questionnaire contains 9 questions to evaluate the subjective perception of the ankle joint during different types of daily activities, such as walking, running, going up and down stairs, and jumping. The total score was 0 ~ 30, and 24 or below suggested an unstable ankle score; (c) they had suffered at least one severe ankle sprain with more than one day of limited mobility, as well as another sprain after the initial injury, with no recent acute sprain within the past three months; (d) they were between the ages of 18 and 25 yrs; and (e) their results from the anterior drawer test and talar tilt test were negative.

The following individuals were excluded from the study: (a) those who had previously undergone surgery or suffered a lower limb fracture; (b) those who had observable skeletal abnormalities in their foot or ankle; (c) those exhibiting mechanical instability or acute pathological symptoms in their lower extremities; and (d) those who had used or received interventional foot orthosis treatment. The participants’ characteristics are displayed in Table 1.

**Table 1:** Physical characteristics of the participants with FAI

Variables	Male	Female
Number/Affected limb	8 (6 right/2 left)	10 (5 right/5 left)
Age (years)	20.9 ± 1.6	20.3 ± 1.3
Height (cm)	176.1 ± 5.4	159.6 ± 3.9
Mass (kg)	68.2 ± 6.7	56.7 ± 10.5
BMI (kg/m^2^)	22.0 ± 2.1	22.1 ± 3.9
CAIT score	18.6 ± 3.2	20.6 ± 1.7

*BMI *Body mass index, *CAIT *Cumberland Ankle Instability Tool

The study was granted ethical approval by the Ethics Committee of Honghui Hospital Affiliated to Medical College of Xi’an Jiaotong University (NO. 202,207,008). All subjects in the study participated voluntarily and signed an informed consent form for the protocol.

### Materials and instrumentations

**Fig. 1:**
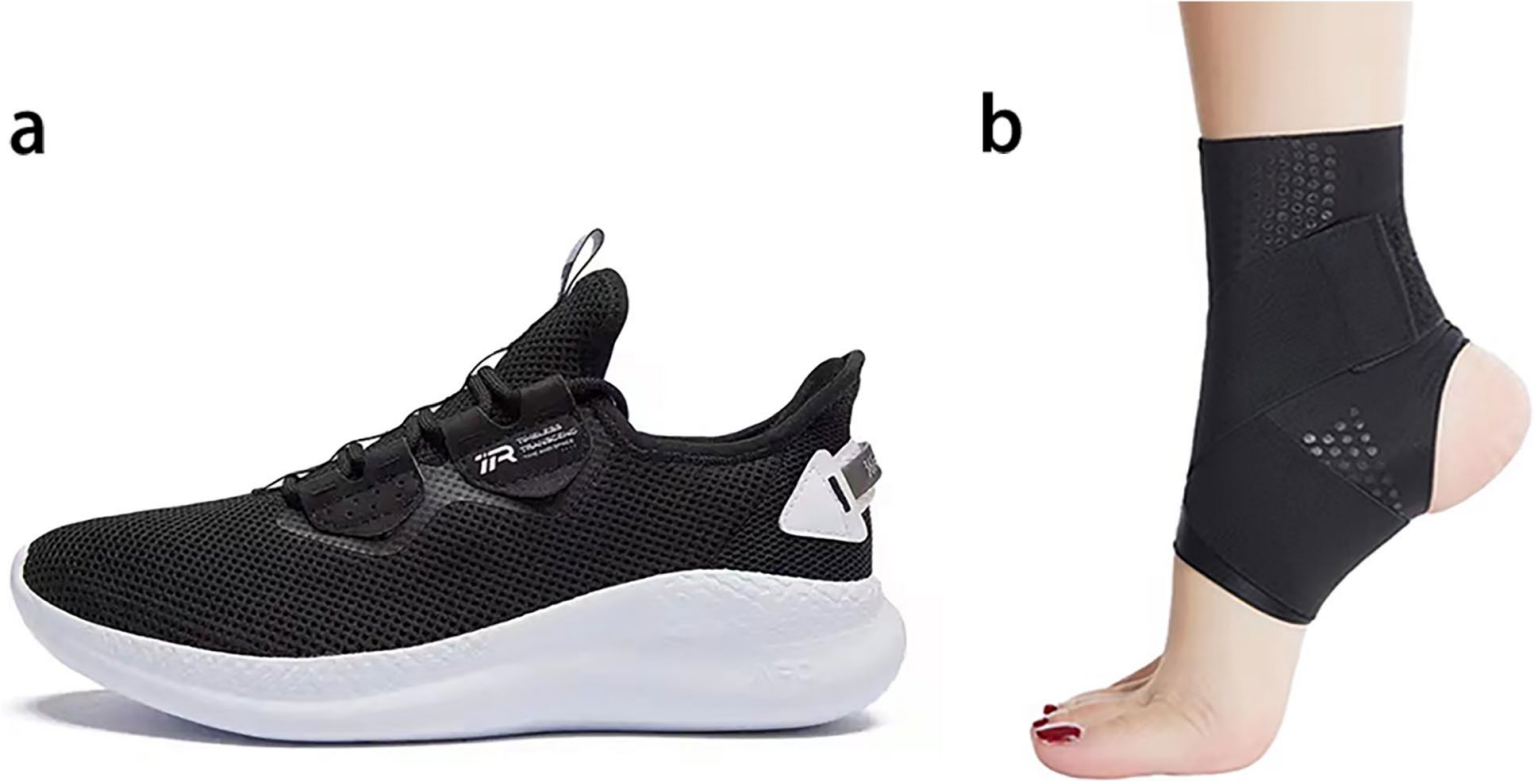
**a** The running shoes and (**b**) the elastic ankle braces in this study

To improve ankle support for participants and eliminate the potential influence of varying types of footwear on the results, this study utilized standardized footwear during testing. It has been demonstrated that athletic shoes with wide soles and sturdy wrapping surfaces enhance the stability of the human body [19]. With this in mind, we used standardized footwear (361° CO. Ltd., Xiamen, China) in the same design for both men and women (Fig. 1a) as the test shoes. The sole is broad, and the upper surface is made of lightweight woven material, providing strong wrapping around the ankle. The test socks were regular knitted cotton socks.

In this study, participants wore elastic cross-band ankle braces (Kante Haiyue, Yangzhou, China) (Fig. 1b). These braces are made of nylon and Spandex, featuring a winding cross-band pattern, and are equipped with Velcro for secure attachment at the end of the band. The brace tension can be easily adjusted, and the heel region is open.

Three types of insoles were evaluated: smooth insoles (control), insoles with 1-mm protrusions, and insoles with 2-mm protrusions. The three types of insoles had similar characteristics except for the varying heights of the protrusions. They were made from EVA material (with a shore value of C50, as displayed in Table 2). For the customization of the custom-made insoles, full-foot scanning was carried out using a 3D foot scanner (Upod-s, Carotec Ltd. Guangzhou, China) in a neutral, non-weight-bearing position. The insole design software Insole CAD (Version 5.4.0, Vismach Technology Ltd. Wuhan, China) was utilized to design full foot arch support orthopedic insoles according to each participant’s foot shape. Therefore, these customized insoles could fit well onto the plantar surface of the participants’ feet to provide appropriate arch and heel support. A 3D carving machine was then used to carve the insoles. Kinetic data during landing tasks were collected using the Kistler 3D force plate (9287B, Switzerland) with a sampling frequency of 1000 Hz.

**Table 2:**
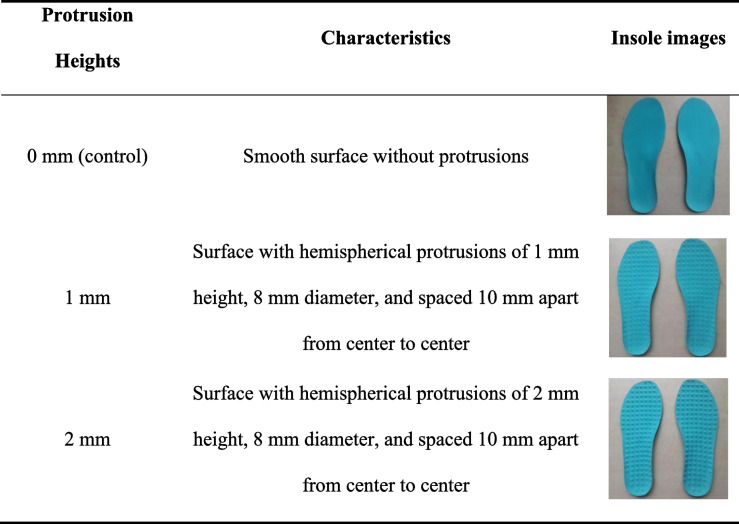
Characteristics of the insoles in this study

### Procedures

During the experiment, the researchers provided the participants with a comprehensive explanation of all experimental protocols and safety measures. The participants wore experimental shoes and familiarized themselves with the test procedures. To ensure safety, all participants underwent three single-leg landing trials to confirm that they could complete the tasks safely. The test conditions consisted of six combinations (3 types of insoles × 2 types of ankle braces). The researchers randomized the test orders beforehand and then informed the participant of his/her specific test orders on the day. The participants were requested to perform three trials for each test condition during the experiment.

**Fig. 2:**
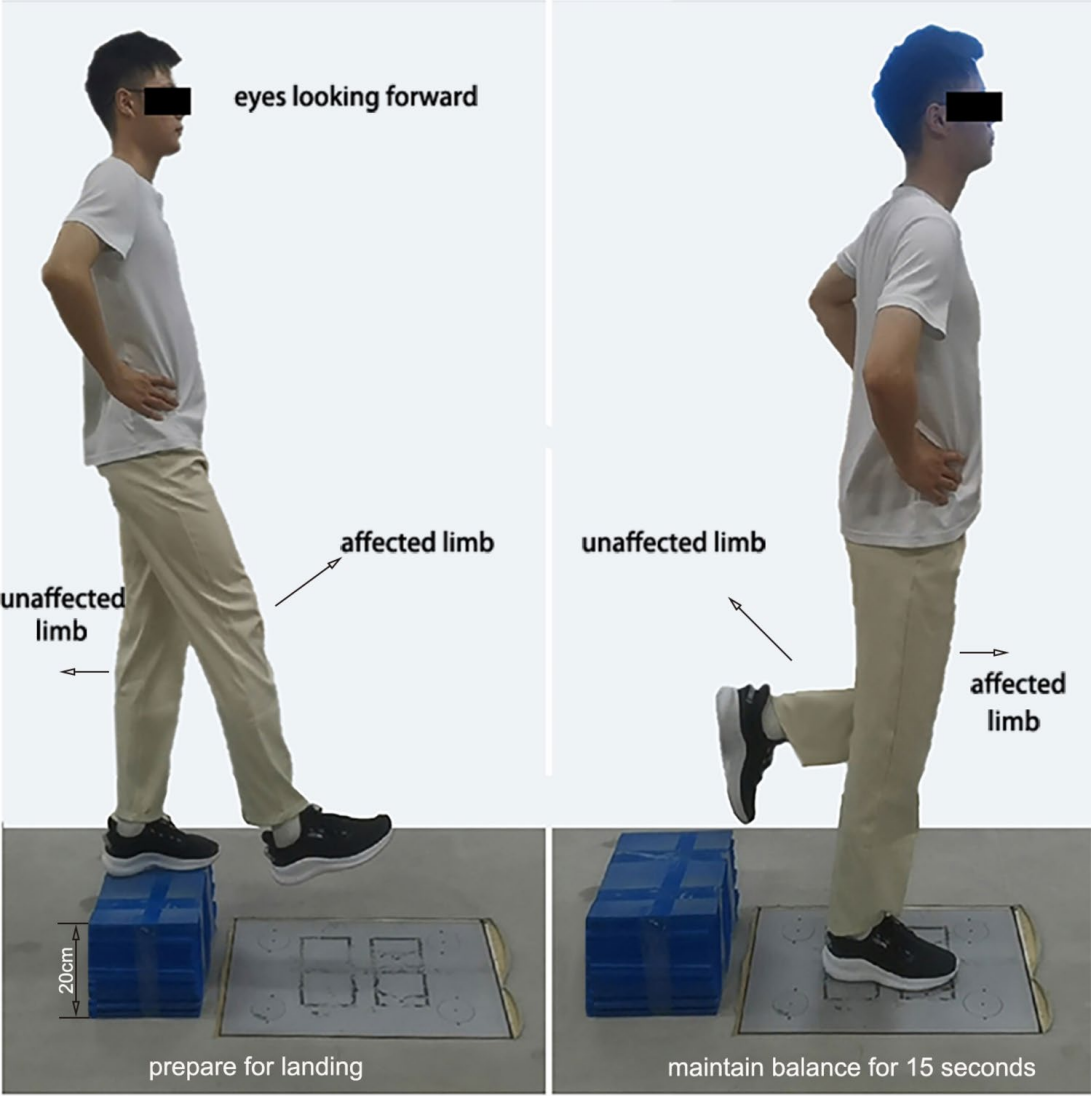
Single-leg landing test without initial velocity

The dynamic stability of the participants was assessed using a single-leg landing task protocol [14, 20] (Fig. 2). The testing platform was positioned 20 cm above the ground and 5 cm away from the edge of the force plate. Participants were directed to stand on the raised platform with their arms on the waist and facing forward. Upon receiving the start signal, the participants dropped toward the center of the force plate. Participants were instructed to drop from the raised platform without an initial vertical velocity. This was visually checked to confirm that the participants did not jump up before leaving the raised platform. After landing, only one limb (the affected limb) supported their weight, while the nonaffected limb was lifted up in the air. Participants were instructed to maintain an upright posture with their eyes open and gaze forward, hands at their waist, and the nonaffected thigh and calf vertically aligned. We required the participants to maintain a single-leg stance for 15 s after landing because this duration has been used in previous studies [15], and it allows us to assess both the initial impact and postural recovery phases of landing. To minimize the effect of fatigue, participants were provided with a 2-minute interval between each landing trial. The researcher closely monitored the procedure to ensure the participant’s safety.

To ensure the validity of the test, the test was repeated if any of the following occurred during: (a) Hands were not naturally positioned at the waist; (b) The nonaffected limb (nonsupporting side) made contact with the ground; (c) The affected limb (supporting side) failed to maintain balance, resulting in the nonaffected limb touching the ground within 15 s of landing; (d) Initial velocity was present at the start of landing (by visual observation); or (e) landing with the nonaffected limb on the ground. In such cases, it was necessary to repeat the test to obtain valid data.

Participants were instructed to subjectively evaluate both stability and comfort of their current condition using two separate 100-mm visual analog scales (VAS) following the landing stability test. The left endpoint of the scale represented “extremely poor”, while the right endpoint represented “extremely good”. Participants were instructed to rate the stability and comfort of various shoe insoles and ankle brace conditions by marking a vertical line at the appropriate position on the scale. The distance from the left endpoint to the marked line represented the score of the respective condition.

### Data processing

Several indices were calculated from the ground reaction force data to indicate dynamic postural stability using a custom-written program. Data during the initial 3 s after landing were analyzed. The indices calculated included the anterior-posterior stability index (APSI), mediolateral stability index (MLSI), and vertical stability index (VSI), which reflect the stability in the anterior-posterior, mediolateral and vertical directions, respectively. The dynamic postural stability index (DPSI) is an extensive evaluation computed by amalgamating the ground reaction force in all three directions, reflecting the comprehensive stability of the human body [21]. Higher APSI, MLSI, VSI and DPSI values correspond to decreased dynamic stability, whereas lower values indicate enhanced stability [22].

1 $$\text{A}\text{P}\text{S}\text{I}=\sqrt{\frac{\sum {\left(0-GRFy\right)}^{2}}{Number\,of\text{ data points}}}{\div}Body\text{ Weight}$$

2 $$\text{M}\text{L}\text{S}\text{I}=\sqrt{\frac{\sum {\left(0-GRFx\right)}^{2}}{Number\,of\text{ data points}}}{\div}Body\text{ Weight}$$

3 $$\text{V}\text{S}\text{I}=\sqrt{\frac{\sum {\left(Body\text{ }\text{Weight}-GRFz\right)}^{2}}{Number\,of\text{ }\text{data points}}}{\div}Body\text{ Weight}$$

4 $$\text{D}\text{P}\text{S}\text{I}=\sqrt{\frac{\sum {\left(0-GRFx\right)}^{2}+\sum {\left(0-GRFy\right)}^{2}+\sum {\left(Body\,Weight-GRFz\right)}^{2}}{Number\,data\,of\,points}}{\div}Body\,Weight$$

### Statistical analysis

In this study, SPSS (version 21.0, SPSS, NY, USA) was utilized for data analysis. For hypothesis testing, a two-way repeated measures analysis of variance (ANOVA) was conducted to investigate the effects of textured insoles and ankle braces on the dependent variables. If the interaction between the two factors (presence/absence of ankle brace and textured insoles) was significant, a simple effect analysis was used to compare different insoles with and without wearing the ankle brace. Otherwise, the main effect analysis was performed to analyze the impact of the two factors on the dynamic stability of FAI patients [23]. A Tukey correction was used to adjust the significance level of post hoc tests, with a significance level of alpha set at 0.05. The effect size was calculated using partial eta-squared (*η*^*2*^_*p*_), which indicates the proportion of variance explained by each factor or interaction. where *η*^*2*^_*p*_ ≥ 0.01 is a small effect, *η*^*2*^_*p*_ ≥ 0.06 is a medium effect and *η*^*2*^_*p*_ ≥ 0.14 is a large effect [24].

## Results

### Dynamic stability

**Fig. 3:**
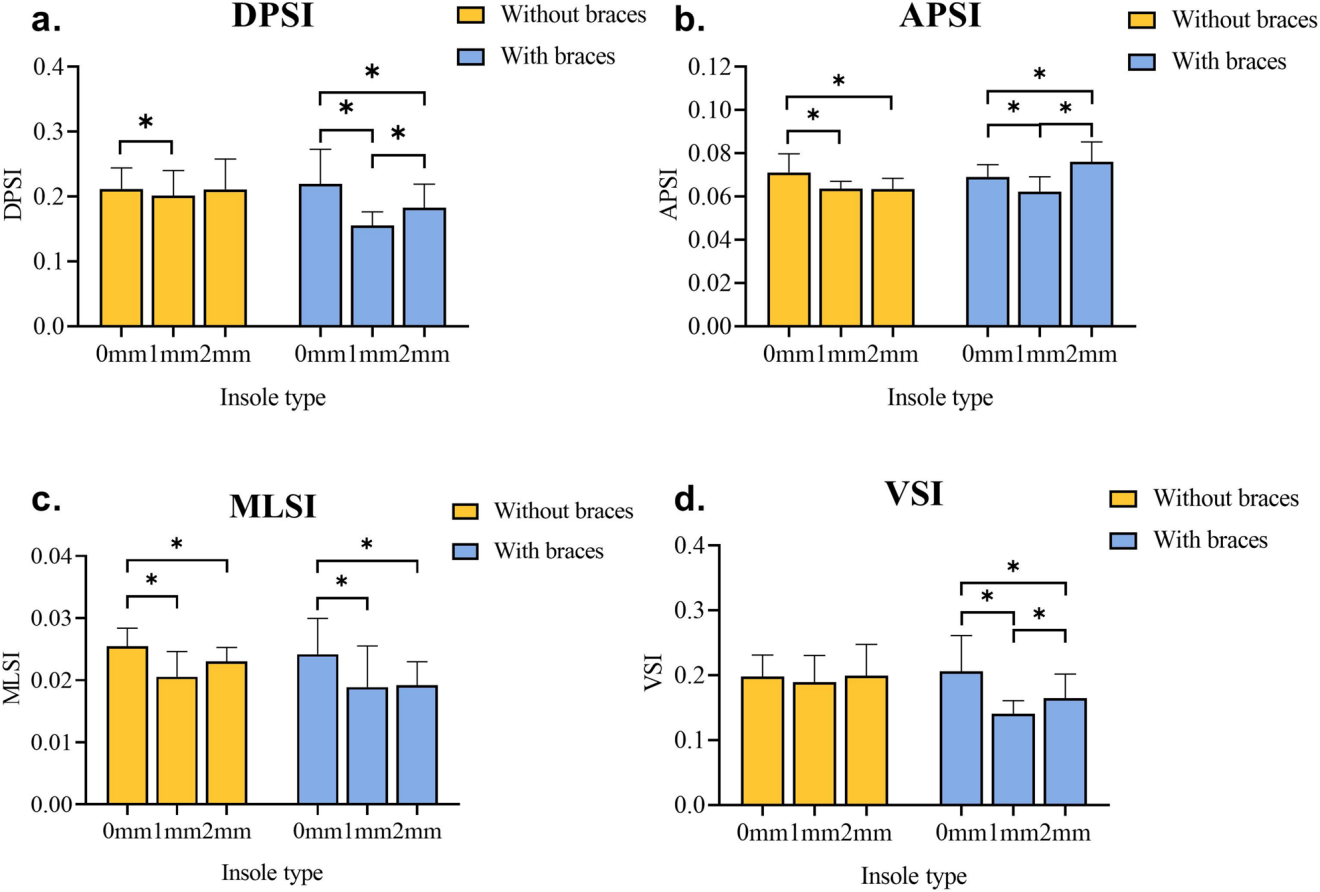
Dynamic stability index in different insoles and ankle brace conditions. Notes: 0 mm, 1 mm, and 2 mm represent insoles with protrusion heights of 0 mm, 1 mm, and 2 mm, respectively, and * represents *P* < 0.05. Figures **a**, **b**, **c**, and **d** are the stability indices in the overall, anterior-posterior, mediolateral, and vertical directions. DPSI: dynamic postural stability index, APSI: anterior-posterior stability index, MLSI: mediolateral stability index, VSI: vertical stability index

Table 3 shows a significant interaction with a large effect between textured insole type and ankle brace for DPSI (*F*_(1.221,20.760)_ = 5.270, *P* = 0.026, *η*^*2*^_*p*_ = 0.237), APSI (*F*_(1.168,19.860)_ = 20.200, *P* < 0.001, *η*^*2*^_*p*_ = 0.543), and VSI (*F*_(1.266,21.510)_ = 5.613, *P* = 0.021, *η*^*2*^_*p*_ = 0.248). Simple effect analysis revealed that textured insoles with 1-mm protrusion had smaller DPSI than 0-mm protrusions without an ankle brace (*P* = 0.014), and textured insoles with 1-mm and 2-mm protrusions had smaller APSI than 0 mm without an ankle brace (*P* = 0.012 and *P* = 0.021, respectively). With an elastic ankle brace, textured insoles with 1 mm protrusion had smaller DPSI (*P* < 0.001, *P* = 0.007), APSI (*P* = 0.003, *P* < 0.001), and VSI (*P* < 0.001, *P* = 0.017) than those with no and 2-mm protrusions. Participants showed smaller DPSI, APSI, and VSI with ankle braces than without (*P* <0.05, Fig. 3).

**Table 3:** Dynamic stability index in different textured insoles and ankle brace conditions

Variables		Protrusion Heights			Insole Type			Ankle Brace			Interaction		
		0 mm	1 mm	2 mm	***F***	***P***	***η***^***2***^_***p***_	***F***	***P***	***η***^***2***^_***p***_	***F***	***P***	***η***^***2***^_***p***_
DPSI	Without braces	0.212±0.034	0.201±0.039^a^	0.211±0.048	7.849	**0.009**	0.316	7.759	**0.013**	0.313	5.270	**0.026**	0.237
	With braces	0.220±0.056	0.156±0.022^a^	0.183±0.039^ab^									
APSI	Without braces	0.071±0.010	0.064±0.004^a^	0.064±0.006^a^	14.980	**<0.001**	0.468	5.396	**0.033**	0.241	20.200	**0.001**	0.543
	With braces	0.069±0.007	0.062±0.007^a^	0.076±0.010^ab^									
MLSI	Without braces	0.025±0.004	0.021±0.005^a^	0.023±0.003^a^	22.650	**<0.001**	0.571	4.473	**0.050**	0.208	0.559	0.555	0.032
	With braces	0.024±0.008	0.019±0.007^a^	0.019±0.005^a^									
VSI	Without braces	0.198±0.035	0.189±0.042	0.199±0.049	7.425	**0.010**	0.304	9.092	**0.008**	0.348	5.613	**0.021**	0.248
	With braces	0.206±0.057	0.141±0.022^a^	0.165±0.039^ab^									

0 mm, 1 mm, and 2 mm represent insoles with protrusion heights of 0 mm, 1 mm, and 2 mm, respectively. The subscripts ‘a’ and ‘b’ denote significant differences from textured insoles with 0-mm and 1-mm protrusions, respectively. *DPSI *Dynamic postural stability index, *APSI *Anterior-posterior stability index, *MLSI *Mediolateral stability index, *VSI V*ertical stability index, ***η***^***2***^_***p***_: partial eta-squared. *P* values marked with bold indicate statistically significant differences. Effect size (partial eta-squared) was interpreted as small (***η***^***2***^_***p***_ ≥ 0.01), medium (***η***^***2***^_***p***_ ≥ 0.06), or large (***η***^***2***^_***p***_ ≥ 0.14)

There was no significant interaction between textured insoles and ankle braces in MLSI (*F*_(1_._748,29.720)_ = 0.559, *P* = 0.555, *η*^*2*^_*p*_ = 0.032). The main effect analysis showed that MLSI was significantly lower for FAI patients wearing elastic ankle braces than for those not wearing elastic ankle braces (*F*_(1,17)_ = 4.473, *P* = 0.050, *η*^*2*^_*p*_ = 0.208), with a large effect. Textured insoles had a significant main effect, with 1 mm (*P* < 0.001) and 2 mm (*P* = 0.006) protrusions having lower MLSI than 0 mm, but no significant difference was observed between 1 and 2 mm (*P* = 0.550, Table 3).

### Subjective perception evaluation

The ankle brace and insole type did not interact in perceived stability (*F*_(1.843, 31.330)_ = 1.102, *P* = 0.341, *η*^*2*^_*p*_ = 0.061, Table 4). FAI patients showed better-perceived stability during landing when wearing ankle braces than those without (*F*_(1,17)_ = 13.140, *P* = 0.002, *η*^*2*^_*p*_ = 0.436), with a large effect. Textured insoles significantly affected perceived stability (*F*_(1.404, 23.870)_ = 8.446, *P* = 0.004, *η*^*2*^_*p*_ = 0.332) with a large effect, with 2 mm protrusion insoles being more stable than 1 mm (*P* = 0.002) and 0 mm (*P* = 0.002) conditions.

**Table 4:** Subjective stability and comfort scores in different textured insoles and ankle brace conditions

Variables	Conditions	Protrusion Heights			Insole Type			Ankle Brace			Interaction		
		0 mm	1 mm	2 mm	*F*	*P*	*η*^*2*^_*p*_	*F*	*P*	*η*^*2*^_*p*_	*F*	*P*	*η*^*2*^_*p*_
Stability(mm)	Without braces	43.9 ± 18.3	48.2 ± 16.5	53.1 ± 17.9^ab^	8.446	**0.004**	0.332	13.140	**0.002**	0.436	1.102	0.341	0.061
	With braces	54.0 ± 14.8	58.3 ± 16.3	68.6 ± 14.5^ab^									
Comfort(mm)	Without braces	58.6 ± 15.0	55.9 ± 17.9	50.1 ± 13.6	0.006	0.987	< 0.001	2.294	0.148	0.119	8.629	**0.002**	0.337
	With braces	56.4 ± 18.4	59.6 ± 14.9	65.6 ± 16.7									

0 mm, 1 mm, and 2 mm represent insoles with protrusion heights of 0 mm, 1 mm, and 2 mm, respectively. The subscripts ‘a’ and ‘b’ denote significant differences from textured insoles with 0-mm and 1-mm protrusions, respectively, ***η***^***2***^_***p***_: partial eta-squared. *P* values marked with bold indicate statistically significant differences. Effect size (partial eta-squared) was interpreted as small (***η***^***2***^_***p***_ ≥ 0.01), medium (***η***^***2***^_***p***_ ≥ 0.06), or large (***η***^***2***^_***p***_ ≥ 0.14)

There was a significant interaction between textured insoles and ankle braces in perceived comfort (*F*_(1.781, 30.270)_ = 8.629, *P* = 0.002, *η*^*2*^_*p*_ = 0.337, Table 4) with a large effect. Simple effect analysis showed that wearing an ankle brace was more comfortable than not wearing an ankle brace for textured insoles with 2-mm protrusions (*P* = 0.005). However, there was no significant difference in perceived comfort between wearing an ankle brace and not wearing an ankle brace for textured insoles with 0-mm and 1-mm protrusions (*P* > 0.05).

## Discussion

The purpose of this study was to determine the effects of textured insoles and elastic ankle braces on dynamic stability in patients with FAI and to identify the optimal design parameters for enhancing dynamic postural stability in these patients. The results of this study demonstrate that the use of textured insoles and elastic ankle braces can lead to an improvement in dynamic stability in patients with FAI.

### Ankle braces and dynamics stability

The results of the present study support Hypothesis 1 that using elastic ankle braces would enhance the dynamic stability of individuals with FAI compared to the absence of ankle braces. These findings align with those reported by Hadadi et al. [2], who demonstrated that using elastic or semi-rigid ankle braces after four weeks of intervention improved dynamic balance in 60 patients with CAI. In the current study, the use of elastic ankle braces with cross bands was employed. The cross-band design of the braces provides compression and security to the soft tissue around the ankle joint, thereby providing mechanical support and reducing excessive inversion and eversion movement in the ankle joint [2]. Additionally, the strong wrapping of the ankle braces enhances proprioception by stimulating the mechanoreceptors in the skin around the ankle joint and elevating motor neuron excitability in the muscle, thereby reducing instability in patients with FAI [2].

### Textured insoles with protrusions and dynamics stability

Hypothesis 2 of this study was that participants would experience improved dynamic stability when wearing textured insoles with higher protrusions would be more effective than those with lesser protrusions. The results partially support Hypothesis 2, as the dynamic stability of patients with FAI was better when they wore textured insoles than when they wore smooth insoles. This improvement may be attributed to the increased tactile input on the plantar surface provided by the hemispherical convexity and arch support of the textured insoles, which also stimulate plantar skin mechanoreceptors by stretching or indenting the skin [8]. Furthermore, insoles with a 1 mm protrusion height showed superior dynamic stability compared to those with a 2 mm protrusion height. The different responses between the 1-mm and 2-mm protrusion heights may be due to the higher protrusions exceeding the optimal sensory threshold for optimal stability, leading to poorer dynamic stability. This speculation is based on the study of Paillard et al. [10], who reported that postural balance was more effectively improved when plantar stimulation was administered close to the sensory threshold (between 90% and 110% of the sensory threshold) than when it was given outside this range (at 70% and 130% of the sensory threshold). In light of these results, future studies should further investigate the effect of textured insoles with different shapes and protrusion heights on stability to better understand the underlying mechanisms of improvement. It should be noted that we did not measure the sensory threshold or plantar stimulation in this study, and therefore our explanation is based on speculation. Further research is needed to quantify the plantar stimulation provided by different textured insoles and its relationship with postural balance.

### Combined effects of textured insoles and elastic ankle braces

Hypothesis 3 of the present study was that using both textured insoles and elastic ankle braces together would result in enhanced dynamic stability compared with using textured insoles or elastic ankle braces alone. This hypothesis was supported, as the results demonstrated a significant interaction between the type of ankle brace and insole, which indicated that the two factors mutually influence each other. It was observed that patients with FAI exhibited improved dynamic stability when wearing a combination of textured insoles and elastic ankle braces compared to utilizing either textured insoles or ankle braces alone. This finding is consistent with the study of Haddadi et al. [12], who investigated the impact of soft ankle support, custom-molded foot orthoses, and the combined mechanism of ankle support on the dynamic stability of patients with ankle instability. The authors reported that combined mechanism ankle support positively affected dynamic stability as measured by the star excursion balance test.

The combination of textured insoles and elastic ankle braces improved dynamic stability in patients with FAI, possibly through the interaction of the two mechanisms of action. The textured insoles provide enhanced motion control [25], with a heel cup design that maintains the hindfoot in a natural neutral position and medial arch support that reduces subtalar joint rotation [26]. Additionally, the combination of insoles and ankle braces may limit the excessive range of motion of the ankle joint, which is a risk factor for ankle sprains [27]. In terms of enhanced perception, the combination of textured insoles and elastic ankle braces enhances the function of both position receptors in the ankle and plantar regions, increases the signals transmitted to mechanoreceptors and skin receptors, and enhances proprioceptive sensitivity through sensory reweighting [28]. Furthermore, this combination improves kinesthetic awareness and the ability of patients with FAI to adjust their posture in an unstable state [29].

One interesting finding of our study was that there was no interaction effect between textured insoles and elastic ankle braces on MLSI, which reflects the mediolateral stability of the ankle joint. This suggests that textured insoles and elastic ankle braces have independent effects on MLSI and that their combined use does not enhance or diminish their individual effects. A possible explanation for this finding is that textured insoles and elastic ankle braces may affect different aspects of the sensorimotor system contributing to mediolateral stability. Textured insoles may primarily stimulate plantar cutaneous receptors and enhance somatosensory feedback from the foot [25], while elastic ankle braces may mainly increase joint stiffness and proprioception by compressing the soft tissue around the ankle joint [21]. Therefore, their effects may not be additive or synergistic but rather complementary. Further research is needed to explore the underlying mechanisms of how textured insoles and elastic ankle braces influence the sensorimotor system and mediolateral dynamic stability.

Overall, the results of this study suggest that the combination of textured insoles and elastic ankle braces is more effective in improving dynamic stability in patients with FAI than utilizing either textured insoles or elastic braces alone.

### Perceived stability and comfort

Lastly, it was hypothesized that ankle braces and textured insoles could improve perceived stability and comfort during single-leg landing. The results showed that textured insoles and braces contributed to improved subjective stability compared to smooth insoles and the absence of braces. However, no interaction effect was evident for perceived stability, indicating that the effects of textured insoles and ankle braces were independent rather than synergistic. These findings were consistent with the results of De Ridder et al. [7], who investigated the impact of foot taping on dynamic postural stability in patients with CAI during jumping landing movements. De Ridder et al. [7] found that taping significantly improved subjective stability perception. The underlying mechanism responsible for these outcomes is speculated to involve textured insoles promoting balance by stimulating the soles of the feet to enhance tactile feedback [30], while ankle braces may improve the afferent feedback of skin receptors, stimulating ankle proprioception [31]. This stimulation elicits a sense of stability, confidence, and psychological comfort, improving perceived stability [32].

This present study has revealed a disparity between subjective and objective stability assessments with respect to textured insoles. The results showed that insoles featuring higher protrusions (2 mm) elicited improved perceived stability compared to those with lower protrusions (1 mm). However, the biomechanical results of the single-leg landing tasks indicated that textured insoles with lower protrusion height (1 mm) provided better stability than those with higher protrusion height (2 mm). This discrepancy can likely be attributed to the fact that the stimulation of the plantar surface provided by 1 mm textured insoles is close to the perception threshold of the plantar, leading to sensory adaptation and decreased tactile perception [5]. On the other hand, the greater stimulation offered by higher protrusion textures may have stimulated the plantar more effectively. Thus, even though tactile perception may be weakened through adaptation, continuous stimulation still contributes to an improved perception of stability, confidence, and peace of mind [33]. As a result, higher protrusions of textured insoles were found to result in a better subjective stability assessment. However, this increased foot stimulation may exceed the optimal plantar stimulation threshold, resulting in poorer objective stability for the higher protrusion texture insoles.

In terms of subjective comfort, we found a significant interaction effect between insole type and ankle brace on the subjective comfort ratings of the participants. This indicates that the perceived comfort of wearing different insoles was influenced by whether they wore an ankle brace. Specifically, we observed that the participants rated the 2-mm insoles as more comfortable when they wore an ankle brace than when they did not, while there was no difference in perceived comfort with and without an ankle brace when FAI patients wore insoles with protrusion heights of 0 and 1 mm. A possible explanation for this finding is that the ankle brace provided a wrapping effect on the plantar surface, which isolated the high-protrusion insoles from direct contact with the skin, thereby reducing the discomfort caused by the excessive stimulation of the plantar mechanoreceptors. However, when the participants wore the low-protrusion insoles, the ankle brace may not have affected the comfort level, as the insoles already provided a moderate and appropriate stimulation of the plantar surface. Therefore, our results suggest that the optimal combination of insole type and ankle brace for subjective comfort may depend on the individual preferences and sensitivity of FAI patients.

### Limitations

This study has some inherent limitations that should be noted. First, the sample ranged in age from 18 to 25 years, which may limit the generalizability of the findings to other age groups, such as children and older adults. Additionally, the participants in the study were within the healthy weight range, so caution should be exercised when extrapolating the results to overweight or obese individuals. Furthermore, the investigation was limited to the immediate effects of textured insoles with varying protrusion heights in the presence and absence of ankle braces, and the study only included two types of textured insoles. Consequently, the broader effects of textured insoles and their long-term impact remain unexplored. Additionally, this study did not delve into the differences in kinematic parameters and muscle activity. Further studies should also consider analyzing lower limb kinematics and muscle activity parameters to obtain a more comprehensive understanding of the effect of foot orthoses on the stability of FAI patients. Last but not least, exploring other highly convex textured insoles may reveal more efficacious approaches for managing FAI.

## Conclusions

This study indicated that using textured insoles in conjunction with elastic ankle braces can enhance dynamic stability in patients with FAI. Specifically, the combination of textured insoles featuring 1-mm protrusions in conjunction with elastic ankle braces demonstrated the most pronounced improvement in dynamic stability compared with using either intervention alone. The stimulation of textured insoles featuring 1-mm protrusions may be close to the optimal sensory threshold, and the combination with elastic ankle braces may further stimulate skin mechanoreceptors and improve proprioception. It is therefore recommended that FAI patients adopt textured insoles with 1-mm protrusions in conjunction with an elastic ankle brace to enhance their stability and to mitigate the risk of ankle sprains.

## Data Availability

The datasets analyzed during the current study are available from the corresponding author upon reasonable request.

## Abbreviations

FAI: Functional ankle instability
BMI: Body mass index
CAIT: Cumberland ankle instability tool
APSI: Anterior-posterior stability index
MLSI: Mediolateral stability index
VSI: Vertical stability index
DPSI: Dynamic postural stability index

## References

[1] Zhang R, Qi Q, Song W, Chen Y (2022). Predicting the success of multimodal rehabilitation in chronic ankle instability based on patient-reported outcomes. BMC Musculoskelet Disord.

[2] Hadadi M, Haghighat F, Mohammadpour N, Sobhani S (2020). Effects of Kinesiotape vs Soft and Semirigid Ankle Orthoses on Balance in patients with chronic ankle instability: a Randomized Controlled Trial. Foot Ankle Int.

[3] Ziaei Ziabari E, Haghpanahi M, Razi M, Lubberts B, Ashkani-Esfahani S, DiGiovanni CW (2022). The Effects of Chronic Ankle instability on the Biomechanics of the Uninjured, Contralateral Ankle during Gait. Orthop Surg.

[4] Kim J, Kang S, Kim SJ (2022). A smart insole system capable of identifying proper heel raise posture for chronic ankle instability rehabilitation. Sci Rep.

[5] He Z, Ye D, Liu L, Di CA, Zhu D (2022). Advances in materials and devices for mimicking sensory adaptation. Mater Horiz.

[6] Ke XH, Huang DB, Li YY, Li XM, Guo JH, Guo MM (2022). Effects of 12 weeks of Tai Chi Chuan intervention on the postural stability and self-reported instability in subjects with functional ankle instability: study protocol for a randomized controlled trial. Front Neurol.

[7] De Ridder R, Willems TM, Vanrenterghem J, Roosen P (2015). Effect of a home-based Balance Training Protocol on dynamic postural control in subjects with chronic ankle instability. Int J Sports Med.

[8] Hadadi M, Ebrahimi I, Mousavi ME, Aminian G, Esteki A, Rahgozar M (2017). The effect of combined mechanism ankle support on postural control of patients with chronic ankle instability. Prosthet Orthot Int.

[9] Abbasi F, Bahramizadeh M, Hadadi M (2019). Comparison of the effect of foot orthoses on Star Excursion Balance Test performance in patients with chronic ankle instability. Prosthet Orthot Int.

[10] Paillard T (2021). Sensory electrical stimulation and postural balance: a comprehensive review. Eur J Appl Physiol.

[11] John C, Stotz A, Gmachowski J, Rahlf AL, Hamacher D, Hollander K (2020). Is an Elastic Ankle support effective in improving Jump Landing Performance, and static and dynamic balance in young adults with and without chronic ankle instability?. J Sport Rehabil.

[12] Hadadi M, Abbasi F (2019). Comparison of the Effect of the combined mechanism ankle support on static and dynamic postural control of chronic ankle instability patients. Foot Ankle Int.

[13] Lin CC, Chen SJ, Lee WC, Lin CF (2020). Effects of different ankle supports on the Single-Leg lateral Drop Landing following muscle fatigue in athletes with functional ankle instability. Int J Environ Res Public Health.

[14] Fransz DP, Huurnink A, Kingma I, de Boode VA, Heyligers IC, van Dieen JH (2018). Performance on a Single-Legged Drop-Jump Landing Test is related to increased risk of lateral ankle sprains among male Elite Soccer Players: a 3-Year prospective cohort study. Am J Sports Med.

[15] Wikstrom EA, Tillman MD, Smith AN, Borsa PA (2005). A new force-plate technology measure of dynamic postural stability: the dynamic postural stability index. J Athl Train.

[16] Kang H (2021). Sample size determination and power analysis using the G*Power software. J Educ Eval Health Prof.

[17] Hiller CE, Refshauge KM, Bundy AC, Herbert RD, Kilbreath SL (2006). The Cumberland ankle instability tool: a report of validity and reliability testing. Arch Phys Med Rehabil.

[18] Hiller CE, Refshauge KM, Herbert RD, Kilbreath SL (2007). Balance and recovery from a perturbation are impaired in people with functional ankle instability. Clin J Sport Med.

[19] Menant JC, Steele JR, Menz HB, Munro BJ, Lord SR (2008). Effects of footwear features on balance and stepping in older people. Gerontology.

[20] Wang YT, Chen JC, Lin YS (2020). Effects of Artificial Texture Insoles and Foot Arches on improving Arch Collapse in flat feet. Sens (Basel).

[21] Maeda N, Urabe Y, Tsutsumi S, Numano S, Morita M, Takeuchi T (2016). Effect of Semi-Rigid and Soft Ankle Braces on Static and Dynamic Postural Stability in Young male adults. J Sports Sci Med.

[22] Sawkins K, Refshauge K, Kilbreath S, Raymond J (2007). The placebo effect of ankle taping in ankle instability. Med Sci Sports Exerc.

[23] Correll J, Mellinger C, Pedersen EJ (2022). Flexible approaches for estimating partial eta squared in mixed-effects models with crossed random factors. Behav Res Methods.

[24] Lakens D. Calculating and reporting effect sizes to facilitate cumulative science: a practical primer for t-tests and ANOVAs. Front Psychol. 2013;4:863. 10.3389/fpsyg.2013.00863. 10.3389/fpsyg.2013.00863 PMC3840331 24324449

[25] Meyer PF, Oddsson LI, De Luca CJ (2004). The role of plantar cutaneous sensation in unperturbed stance. Exp Brain Res.

[26] Richie DH (2007). Jr. Effects of foot orthoses on patients with chronic ankle instability. J Am Podiatr Med Assoc.

[27] Hubbard TJ, Hertel J (2006). Mechanical contributions to chronic lateral ankle instability. Sports Med.

[28] Bapirzadeh K, Jamali A, Forghany S, Nester C, Tavakoli S, Hemmati F (2014). The effect of three different insoles on balance in people with functional ankle instability. J Foot Ankle Res.

[29] Eils E, Demming C, Kollmeier G, Thorwesten L, Volker K, Rosenbaum D (2002). Comprehensive testing of 10 different ankle braces. Evaluation of passive and rapidly induced stability in subjects with chronic ankle instability. Clin Biomech (Bristol Avon).

[30] Park JH, Benson RF, Morgan KD, Matharu R, Block HJ (2023). Balance effects of tactile stimulation at the foot. Hum Mov Sci.

[31] Zhang Z, Zhang M (2023). Effect of different ankle braces on lower extremity kinematics and kinetics following special-induced fatigue for volleyball players with functional ankle instability. Heliyon.

[32] Han S, Lee H, Son SJ, Hopkins JT (2022). The effects of visual feedback disruption on postural control with chronic ankle instability. J Sci Med Sport.

[33] Lin JZ, Lin YA, Tai WH, Chen CY (2022). Influence of landing in neuromuscular control and ground reaction force with ankle instability: a narrative review. Bioeng (Basel).

